# Meeting Reports

**DOI:** 10.1155/S146392468200011X

**Published:** 1982

**Authors:** F. L. Mitchell, Clive J. Jackson

**Affiliations:** 1 Clinical Research Centre Watford Road Harrow Middlesex HA1 3UJ UK; 2 The Occupational Medicine and Hygiene Laboratories Health & Safety Executive 403 Edgware Road London NW2 6LN UK


Journal of Automatic Chemistry, Volume 4, Number 1 (January–March 1982), pages 41–42

### Meeting Reports

### The XIth International Congress of Clinical Chemistry

Every three years the International Federation of Clinical Chemistry (IFCC) organizes its International Congress—in 1981 it was held in Vienna. There are different views as to the value of such large gatherings of scientists of like mind. The younger end of the profession attend to sit at the feet of the maestros; some take the opportunity to meet colleagues with similar problems and pay scant attention to the scientific programme, assuming that everything of any worth will appear shortly in the literature; some attend associated conferences concerned with administration and organization; some rigorously study the programme to produce their private list of items selected for attendance in order to maximize on the opportunity for learning; some concentrate exclusively on the attendant exhibition and a few use the whole as an excuse to sight-see and have a superb holiday with a magnificent scientific excuse. In Vienna there was ample opportunity to satisfy all such desires, thanks to the now considerable experience of the IFCC Conference Committee.

Although almost every aspect of clinical chemistry was covered, it is pertinent here to concentrate only on areas concerning instruments.

The six distinguished plenary lecturers had a common theme covering problems relating to the development of the relatively new speciality of clinical chemistry in the present changing conditions of medicine. Dr H. Keller of St. Gall, Switzerland, perhaps the one most orientated towards instruments, stressed 'Methodological and instrumental development should not be a monopoly of industrial laboratories. Clinical chemistry laboratories of all sizes should participate more frequently in research projects'.

Of the 23 congress symposia, one covered the application of HPLC in clinical chemistry, one the automation of radioimmunoassay techniques, and another luminescence.

There were six company symposia, in two of which Miles Laboratories discussed their solid-phase chemistry and Kodak introduced their 'Principles of multi-layer film analyses', with performance data on instruments using the system involved.

The very large instrument exhibition gave its usual thorough coverage of items both commonplace and exotic. There were two exhibits displaying principles which were new to Europe at least. Kodak most elegantly displayed the Ektachem 400 utilizing their revolutionary dry phase, layered chemistry system. Coulter presented their DACOS (Discrete Analyser with Continuous Optical Scanning), admittedly ahead of its being fully operational, but showing much promise with its prospects of being able to monitor continuously, all samples undergoing reaction at any one time. Technicon in their usual major display of automatic machines to meet a range of needs, launched the RA1000—a discrete bench-top, random-access analyser capable of performing up to 12 different analyses per sample at an operational rate of 140 tests/h.

The IFCC Expert Panel on Instrumentation presented a timely symposium on 'Acceptable results from small instruments'. With the development of small, cheap, reliable but complex electronics, the maxim 'big is beautiful' is no longer universally applicable in clinical chemistry. During this century, pathology has moved to the central laboratory because to carry it out near the patient has become increasingly less practical and efficient. This trend is now reversing and the Panel decided that a discussion was necessary on the implications of this and whether, in the interests of speed and economy, a certain amount of slippage in precision and accuracy might be acceptable. Dr Free (Elkhart, USA), covered the history of the development of small instruments; Mr Sylvester (Rochester, New York, USA) the statistics defining the clinician's needs; Dr Rinsler (Harrow, UK) the practical implications; Dr Geary (Adelaide, Australia) the criteria for instrument acceptability; Dr Naka (for Professor Okuda, Japan) practical experience in Japan. A lively discussion was led by Dr Young (Rochester, Minnesota, USA) and Dr Eilon-Gerritzen (Rotterdam, The Netherlands). Perhaps predictably, little progress was made under the specific theme of the title of the symposium but much was learnt, aired and discussed. It was very evident that in this area there is considerable scope for future conference debate.

The whole Congress took place in the Hofburg Palace, the home of the Hapsburg Empire, which has been converted into a congress centre with a most satisfying blend of the old and new. In the centre of Vienna and within walking distance of most of the city's attractions, the old palace would go a long way to make any congress a memorable occasion and this one was no exception.

**F. L. Mitchell**
*Clinical Research Centre, Watford Road,*
*Harrow, Middlesex HA1 3UJ, UK*

### Automated analysis—the expanding choice

A joint symposium on the applications of microprocessors and microcomputers in automated analytical chemistry, organized by the Automatic Methods Group and the Scottish Region of the Analytical Division of the Royal Society of Chemistry (RSC), together with the Microcomputer and Microprocessor Subject Group of the RSC, was held at Stirling University from 9 to 11 September 1981.

Three lecture sessions were held—Basic principles and development trends; Data handling; and Computer graphics. In addition, an afternoon and early evening were devoted to a series of concurrent workshop presentations, partially structured to provide a degree of 'hands-on' experience for delegates, and partially open and available for discussion of participants' specific interests.

In the first session on basic principles and development trends, three broadly based papers were presented to provide an in-depth introduction to the use of microprocessors and microcomputers in analytical instrumentation. Dr Stockwell (Plasma-Therm Ltd, London) reviewed the influence of microprocessors on the design and introduction of automation in the analytical chemistry laboratory, drawing on his wide experience gained whilst at the Laboratory of the Government Chemist (London) with the addition of a number of new ideas. Dr Newton (AERE, Harwell, UK) gave an excellent status report on the current development of micros. He paid particular attention to memory, peripheral and processor devices as well as display, data storage and communication technologies. Particularly enlightening was his predictions of future trends in these areas. Finally, Dr Carrick (Kratos Ltd, Manchester, UK) discussed the requirements and problems of interfacing computers to instrumentation. He particularly considered the requirements for, and capabilities of, standard serial and parallel interfaces (RS 232C and IEEE 488) as well as their applications. A wide-ranging discussion then followed covering the development of new computer languages, future developments in printers, the future for personal computers in view of the reduction in size of main-frame computers and the problems of voice recognition.

The session continued with the presentation of nine concurrent workshops on aspects of and applications of microprocessors and microcomputers to analytical instrumentation. Before the individual demonstrations each workshop presenter gave a brief overview of his workshop to the complete audience, who then dispersed to take part in the workshops illustrating many of the points being presented in the formal paper sessions. Data acquisition and handling using micros was the subject of Dr Newton's workshop, whilst Dr Tranter (Glaxo Operations [UK] Ltd, Barnard Castle, UK) covered aspects of data handling and presentation in an HPLC situation. Both Professor Betteridge (University College, Swansea, UK) and Mr Porter (Laboratory of the Government Chemist) dealt with aspects of flow-injection analysis. Mrs Evans-Terlecki (Telmoss Ltd, Welwyn Garden City, UK), Dr Braithwaite (Trent Polytechnic, Nottingham, UK) and Mr Linnet (Health & Safety Executive, Edinburgh, UK) dealt with various aspects of interfacing. Dr Berridge (Pfizer Central Research, Sandwich, UK) demonstrated micro-controlled automated simplex optimization as applied to HPLC, whilst Dr Smith (University of Technology, Loughborough, UK) discussed aspects of graphical display of data.

The second session on data handling was launched by an amusing and thought-provoking paper by Professor Betteridge—on the perennial problem of what to do with the mass of experimental data that can be obtained from an experiment. He concentrated on two basic ideas (1) getting the experiment right, using, for example, simplex optimization routines; and (2) abstracting new forms of information from collected data using cluster analysis (pattern recognition) and factor analysis. He was followed by Dr McLelland (Glasgow Royal Infirmary, UK) who spoke about the control and management of a large automated clinical chemistry department handling a third of a million samples and producing 1·5 M analyses per annum. He discussed the logistics of handling such an amount of data, and the procedures (network analysis) used in optimizing experimental design and organizing the department to permit such a throughput. Dr Berridge then spoke about the applications of a microprocessor to enable linear and simplex optimization of suitable HPLC analytical procedures to be fully automated. Finally, Mr Usher (Unilever Research, Sharnbrook, UK) indicated how, in his laboratory, a Hewlett-Packard microcomputer was used to link a range of analytical instruments to a site computer. He discussed the sample and report-handling procedures used.

Three papers were presented in the third session on computer graphics, the first by Dr Smith who demonstrated some applications of high-resolution graphical display of data using an Apple II microcomputer. Then Dr Tranter discussed the use of an HP85 micro to evaluate and graphically present data obtained from an HP8450 diode-array spectrophotometer used as an HLPC detection system. He emphasized applications involving the detection of hidden or unsuspected components in a mixture. Finally, Mr Aitchison (Heriot-Watt University, Edinburgh, UK) spoke on interactive graphical plotting and indicated how additional information could be obtained from multi-dimensional isometric plots.

In addition to the scientific programme, a range of social events were provided for delegates. These included a reception on the evening before the symposium opened, a Highland dinner and a golf tournament and supper at Gleneagles.

The general feeling was that the symposium had been a success and that the 140 participants had much appreciated the varied events. The large attendance was very gratifying for the organizers, speakers and workshop presenters, although a few problems were apparent in the allocation of participants to the various structured workshops.

**Clive J. Jackson**

*The Occupational Medicine and Hygiene Laboratories, Health & Safety Executive, 403 Edgware Road, London NW2 6LN*

### Swansea Summer School of Automatic Chemical Analysis

The 1982 Swansea Summer School takes computers in automation and laboratory management as its theme. The School is a one-week, intensive course intended to provide analysts from all sectors of industry with the skills needed for laboratory automation. The programme includes a balanced mix of lectures, tutorials and practical classes and, as far as possible, is tailored to participants' specific requirements. Topics to be covered include: automated instrumental analysis, flow injection analysis, automated chromatography, spectroscopy and titrimetry, the systems approach to automation, managing an automated laboratory, microprocessors, fundamentals of computing, advanced computing, analytical quality control.

Lecturers at the 1982 School include Dr R. W. Arndt (Switzerland), Professor D. Betteridge (UK), Dr A. Carrick (UK), Professor M. Bonner Denton (USA), Professor G. Horlick (Canada), Professor D. L. Massart (Belgium), and Professor H. L. Pardue (USA).

The School will be held from 11–16 July 1982 at University College, Swansea, UK.

*Further information from Professor D. Betteridge, Chemistry Department, University College, Singleton Park, Swansea SA2 8PP, UK.*

